# Epidermal Wearable Biosensors for Monitoring Biomarkers of Chronic Disease in Sweat

**DOI:** 10.3390/bios13030313

**Published:** 2023-02-23

**Authors:** Xichen Yuan, Chen Li, Xu Yin, Yang Yang, Bowen Ji, Yinbo Niu, Li Ren

**Affiliations:** 1 School of Mechanical Engineering, Northwestern Polytechnical University, Xi’an 710072, China; 2 Key Laboratory for Space Bioscience and Biotechnology, School of Life Sciences, Northwestern Polytechnical University, Xi’an 710072, China; 3 MOE Key Laboratory of Micro and Nano Systems for Aerospace, Northwestern Polytechnical University, Xi’an 710072, China; 4 Key Laboratory of Flexible Electronics of Zhejiang, Ningbo Institute of Northwestern Polytechnical University, Ningbo 315103, China; 5 Ministry of Education Key Laboratory of Low-Grade Energy Utilization Technologies and Systems, Chongqing University, Chongqing 400030, China; 6 Unmanned System Research Institute, Northwestern Polytechnical University, Xi’an 710072, China

**Keywords:** epidermis, wearable biosensor, sweat, biomarkers, chronic disease, preclinical

## Abstract

Biological information detection technology is mainly used for the detection of physiological and biochemical parameters closely related to human tissues and organ lesions, such as biomarkers. This technology has important value in the clinical diagnosis and treatment of chronic diseases in their early stages. Wearable biosensors can be integrated with the Internet of Things and Big Data to realize the detection, transmission, storage, and comprehensive analysis of human physiological and biochemical information. This technology has extremely wide applications and considerable market prospects in frontier fields including personal health monitoring, chronic disease diagnosis and management, and home medical care. In this review, we systematically summarized the sweat biomarkers, introduced the sweat extraction and collection methods, and discussed the application and development of epidermal wearable biosensors for monitoring biomarkers in sweat in preclinical research in recent years. In addition, the current challenges and development prospects in this field were discussed.

## 1. Introduction

Chronic diseases, including cardiovascular diseases, chronic respiratory diseases, tumors, diabetes, and other diseases, are the leading cause of death throughout the world and account for about 60% of all deaths. Early detection is key to the prevention of chronic diseases. In recent years, people have become increasingly interested in personal health monitoring, management, and personalized medicine, which is expected to improve health and quality of life through the early detection of chronic diseases. The innovative method of using biomedical equipment to provide personalized monitoring, management, and treatment according to the biochemical conditions of each person is expected to revolutionize traditional medical practice [1,2]. Since the 20th century, biochemical monitoring has undergone four technological changes. The earliest changes occurred in the early 20th century when dedicated testing instruments were needed to detect biochemical information in a specialized analytical laboratory. The second wave of technology was at the beginning of the 21st century, namely, point-of-care testing (POCT), which utilized sampling and immediate testing right next to the patient, eliminating complicated specimen processing procedures in laboratories. The third technological change, currently happening right now, is the emergence of wearable devices: a category of portable detection devices that can be directly worn on the epidermis, oculi or oral cavity, or integrated into the user’s clothes or accessories for in situ detection of biomarkers in various body fluids (e.g., sweat, interstitial fluid, tears, and saliva). The latest technological innovation is a fully implantable biochemical sensor, which can be implanted into tissues through minimally invasive techniques for real-time monitoring. For health monitoring and early warning of chronic diseases, epidermal wearable biosensors will be the first choice due to their significant properties of low cost, portability, and sensitivity.

The concept of biosensors was first proposed by Dr. Leland C. Clark in 1962 [3], who defined it as an analytical device used to provide real-time data on one or more biomarkers in a sample. A typical biosensor contains two basic functional units: a “bioreceptor” (for example, the enzyme, antibody, aptamer, etc.), which is responsible for the selective recognition of target analytes; and a physical–chemical sensor (for example, an electrochemical, optical, or mechanical sensor) that converts biological cognitive events into useful signals [4]. There are three key operating processes: sample collection, biochemical reaction, and output quantifiable signals [5]. Biosensors have broad prospects in the wearable applications field due to their high specificity, quick response, portability, and low power consumption [4]. Wearable biosensors can in situ non-invasively measure biomarkers in body fluids to provide real-time physiological and biochemical information, thereby providing sufficient information for health monitoring and even preliminary medical diagnosis, which has attracted widespread attention.

Epidermal biosensors can facilitate the real-time analysis of biomarkers in sweat and have continuous monitoring capabilities for biomedical research, clinical diagnosis, and treatment [6], thereby providing sufficient information for a preliminary medical diagnosis. In recent years, wearable biosensor platforms for the detection of biomarkers in sweat have been widely used in various parts of the body (e.g., the arm, forehead, chest, and back). The concept was proven using a series of important analytes as targets. Through continuous and real-time monitoring of biomarkers related to the wearer’s health condition, we can have an in-depth understanding of their dynamic biochemical processes and provide health information, thereby strengthening the management of their chronic diseases. The epidermal wearable biosensor avoids having to damage the skin by the pierce of a needle during blood sampling, thereby allowing the wearer to use it in daily life [7,8]. At present, wearable biosensors have been manufactured on a variety of substrates, such as textiles, gloves, wristbands, smart bandages, temporary tattoos, etc. [9,10]; therefore, they are convenient and comfortable to wear.

In this review, we first introduced the composition of sweat and then explained the sweat collection and analysis methods. In addition, the applications of epidermal wearable biosensors in health monitoring and early warnings of chronic diseases were summarized, and their challenges and developmental potential were discussed.

## 2. Sweat Characteristics

The epidermis covers most of our bodies; sweat glands are distributed throughout the human body and can be categorized as eccrine, apocrine, and apoeccrine according to their locations, structures, and functions [11,12]. The eccrine sweat gland is the most abundant sweat gland for thermoregulation through the continuous secretion of serous fluid containing various types of solute [13] distributed over nearly the entire body surface. There are approximately 1.6 to 4.0 million glands on the adult body surface, and the average density of the glands varies in different persons and anatomic sites; for example, 64 glands/cm^2^ on the back, 108/cm^2^ on the forearm, 181/cm^2^ on the forehead, and 600 to 700/cm^2^ on the palms and soles [13,14]. Thus, there are more than 100 glands per square centimeter of skin on most of the body surface [6]. Children have the same number of eccrine sweat glands as adults; therefore, the average density of the glands is higher in children than in adults [13,15]. In most climatic conditions, ordinary adults secrete 500–700 mL of hypotonic fluid per day from sweat glands [4]. Sweat is produced by exercise, heat, and emotional stress [16]. The sweat secretion rate of human skin varies with individual differences, exercise status, environmental temperature and humidity changes, and some disease states will increase the sweat secretion rate and amount (e.g., menopausal symptoms, cardiomyopathy, etc.) [17,18]. Therefore, due to the high-density distribution of eccrine sweat glands both in children and adults and the considerable sweat secretion, sweat can be collected non-invasively using a compact epidermal wearable biosensor [19,20,21].

### 2.1. The Components of Sweat

**Figure 1:**
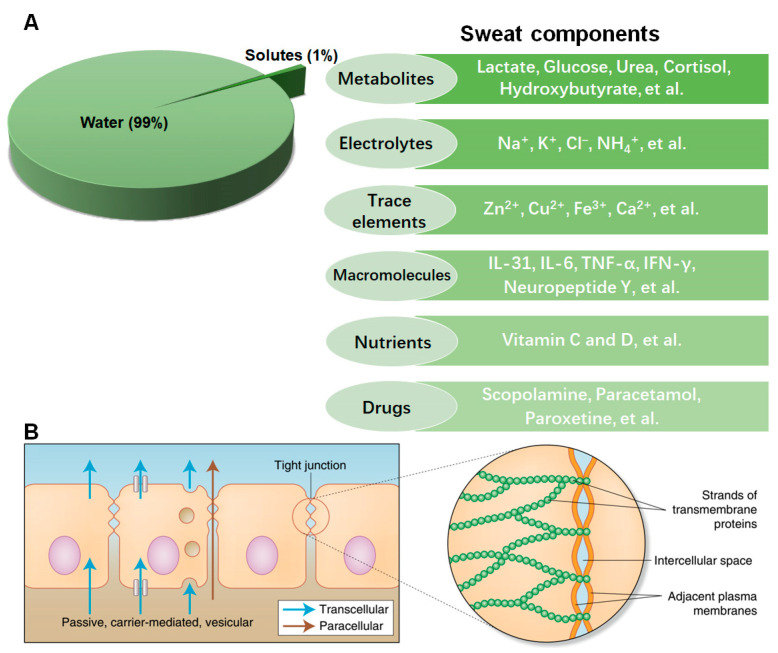
Schematic diagram of (**A**) the sweat components and (**B**) three ways for biomarkers to enter sweat (Reprinted with permission from Ref. [43]. Copyright 2019, Springer). One way is by diffusion through the plasma membrane of capillary endothelial cells; the other is diffusion or advection transport between cells; and the third is transport through cell vesicles.

Sweat is an acidic electrolyte and metabolite-rich liquid [22]. The study of biomarkers in sweat began in 1910 when Embden proved that the amino acid serine exists in human sweat [23]. Since then, there has been an accumulating body of studies aimed at determining the composition of human sweat. Exogenous substances such as ammonia, glucose, chloride, prostaglandins, C12-C22 fatty acids, drugs, and ethanol are separated from sweat [12,24]. Now, studies have confirmed that perspiration is mainly composed of water (99%); metabolites (such as lactate, glucose, urea, cortisol, hydroxybutyrate, etc.) [20,25,26,27,28]; electrolytes (such as Na^+^, K^+^, Cl^−^, etc.) [20,29,30]; trace elements (such as Zn^2+^ and Cu^2+^, Fe^3+^, etc.) [31,32,33]; a small amount of macromolecules (such as cytokines IL-31, IL-6, TNF-α, IFN-γ, neuropeptide Y, etc.) [34,35,36,37,38]; nutrients (such as vitamin C and D) [39,40]; and drugs (such as scopolamine, paracetamol, paroxetine, etc.) [41,42], as shown in Figure 1. Some of them are closely related to health status and can be used as biomarkers for in situ non-invasive monitoring of physiological health status and the genesis and development of chronic diseases and the effectiveness of treatment.

Analytes in the blood are secreted into sweat through the cell barrier. As shown in Figure 1, there are three main ways for solutes to enter sweat: (1) diffusion through the plasma membrane of capillary endothelial cells, (2) diffusion or advection transport between cells, (3) and transport through cell vesicles [43,44]. Sweat analytes are each transported to the sweat in different ways. A deep understanding of the correlations between sweat analytes and health status is extremely important for health monitoring and the development of sweat sensors [16].

#### 2.1.1. Electrolyte Ions and Trace Elements

Studies have confirmed that there are different kinds of ions in sweat, including Na^+^, K^+^, Cl^−^, NH₄^+^, Fe^2+^, Mg^2+^, Ca^2+^, etc. [24,45,46,47,48]. The secretion of Na^+^ is mainly determined by the process of sweat secretion, and there is no direct relation to the Na^+^ level in the blood. The Na^+^ concentration in the blood is 135–145 mM, while the Na^+^ concentration in sweat is tens of mM [24]. While some studies have shown that K^+^ in sweat has nothing to do with the rate of sweat production, and its correlation with blood K^+^ concentration has yet to be confirmed, other studies have found that K^+^ may vary within the blood concentration range [45]. Correspondingly, the concentration of Fe^2+^ in sweat is positively correlated with the concentration of Fe^2+^ in the blood, and the concentration of Fe^2+^ in sweat is hardly affected by the sweat rate, and its secretion is not affected by other ions [46]. In contrast, Mg^2+^ ions do not act in a similar manner. Researchers have also studied the sweat of patients with renal failure, and the results showed that the concentration of Mg^2+^, Ca^2+^, and phosphate in the sweat of patients with renal failure was increased, which indicated a change in the concentration of disease-specific sweat ions [47]. Elevated chloride levels were monitored in patients with infantile cystic fibrosis [47]. Researchers have proved that the water and electrolytes in the sweat of patients with psoriasis are reduced, opening up the field of sweat analysis in dermatology.

#### 2.1.2. Hydrophilic Components

The secretion process of hydrophilic components (glucose, lactate, proteins, cytokines, etc.) depends on active or passive channels. Due to the highly filtering structure formed by the tight junctions of more than 40 different proteins [49], conversion factors related to blood concentration are required.

The entry pathway for sweat glucose is next to the cell. Researchers have also detected that there is a certain correlation between the concentration of glucose in sweat and the concentration of glucose in the blood and surrounding interstitial fluid (ISF) [50,51]. There is a potential development in the detection of sweat glucose for continuous monitoring of the physiological condition of diabetic patients. At present, the number of diabetic patients is gradually increasing, so the research on wearable biosensors is mainly focused on glucose detection.

Moreover, researchers have shown that measuring changes in the concentration of lactate in sweat can be used to detect ischemia [52].

The cytokine concentration in the blood is between pM and nM, while the cytokine concentration in the sweat is less than 0.1% of its concentration in the blood [53]. The protein content in sweat is usually more than 1000 times lower than that in the ISF and the blood [54]. However, from the perspective of biosensing, the degradation rate of proteins and cytokines is very slow, due to the proteases and enzymes that will be highly diluted and will catabolize their target analytes slowly [43].

#### 2.1.3. Lipophilic Components

The lipophilic components secreted into sweat depend on passive diffusion through the skin barrier [12]. The concentration levels of small lipophilic (hydrophobic) molecules, such as cortisol [55], testosterone [43], and drugs (e.g., ethanol [56], levodopa [57], methylxanthine [58], etc.) in sweat is strongly correlated with their concentration levels in blood. 

There are considerable studies on electrolytes, lactic acid, and other metabolites in sweat, while there are fewer studies on trace elements and macromolecules, such as Mg^2+^ and cytokines. This factor is because biomarkers in sweat are transported from the surrounding capillaries to the sweat and can also be produced in the sweat duct, which makes them difficult to reliably correlate with the blood-drug concentration at the same time. On the other hand, it is more meaningful to analyze trace elements and macromolecules in blood. In addition, the correlation between many analytes in sweat and corresponding analytes in blood needs further verification [22,43,45,59,60]. Although this currently means that these analytes cannot be directly related to systemic conditions, they can at least be related to sweat gland movement in response to systemic conditions, so corresponding clinical applications can also be carried out [24]. The clinical verification of sweat analytes is very important for the overall development of sweat sensing, especially for commercial applications. The blood-sweat correlation must be established through in vivo experiments, and the distribution pathway of biomarkers must be fully understood in order to fully realize the potential of wearable sweat sensors.

### 2.2. Sweat Biomarkers of Chronic Disease

Some biomarkers in sweat have been used as the gold standard or an important reference for disease diagnosis, such as sweat Cl^−^ and glucose [30,51,61]. In addition, a lot of components have been confirmed by laboratory studies and are expected to become new-generation disease biomarkers, as shown in Table 1. 

Cystic fibrosis is a life-shortening rare autosomal recessive disease that results in abnormal sweat composition both in newborns and adults, elevated sweat Cl^–^ concentration is the gold standard biomarker for the diagnosis of cystic fibrosis in clinical [30,62,63,64,65]. A sweat Cl^−^ level ≥ 60 mM for all populations (newborns and adults) is the threshold for a definitive diagnosis of cystic fibrosis and a sweat Cl^−^ level < 30 mM indicates that cystic fibrosis is unlikely. In individuals who fall into the intermediate sweat chloride level, 30–59 mM, genetic analysis is required, as shown in Table 1 [30].

Diabetes is a worldwide chronic disease associated with high circulating blood glucose concentrations. Monitoring blood glucose plays an important role in the management of diabetes and the reduction of the risk of serious secondary clinical complications [66]. What is interesting is that healthy humans and diabetic patients show a certain correlation between sweat glucose and blood glucose [51,67], the glucose concentrations are ~100× lower in sweat than in blood [51,61]. Therefore, precisely measuring sweat glucose concentration can estimate blood glucose levels in a non-invasive and convenient way [8,67].

Diabetic ketoacidosis is a condition caused by the accumulation of ketone bodies in patients with hyperglycemia or metabolic acidosis. β-hydroxybutyrate, a dominant physiological ketone detected in sweat and other body fluid can be used as a biomarker of diabetic ketoacidosis [68,69].

Behcet’s disease (BD) is an autoimmune disorder with the serious possibility of blindness; patients often have a special and unpleasant odor, which might be caused by the abnormal composition of their sweat [70]. A metabolomics analysis of BD patients’ sweat revealed that a panel of metabolites can be selected as candidate biomarkers, including l-citrulline, l-pyroglutamic acid, urocanic acid, 2-oxoadipic acid, cholesterol 3-sulfate, and pentadecanoic acid [70]. 

Schizophrenia (SZ) is a severe, chronic, and debilitating brain disorder that is characterized by distortions in thought and perception [71]. Due to the possibility of patient opposition or discomfort related to blood collection, detection through non-invasive sweat biosensors would be a convenient approach. Healthy control and SZ patient sweat sample analyses revealed that five sweat proteins (Annexin-5, Bleomycin hydrolase, Thioredoxin, Caspase-14, and Synaptophysin) were differentially abundant in sweat [72].

Epilepsy is a chronic disease with a sudden abnormal discharge of brain neurons, resulting in transient brain dysfunction. A specific volatile organic compound in sweat, menthone, a newly described human alarm pheromone, may be an early seizure biomarker [73].

Furthermore, some cytokines in sweat were considered to be related to a series of chronic diseases. Interleukine-31 (IL-31) is involved in allergic rhinitis and skin-based autoimmune disorders such as pruritis, alopecia, psoriasis, and atopic dermatitis [34,74]. Interleukin-6 (IL-6) is associated with chronic disease in older adults and is the best predictor of all-cause mortality compared to most other cytokines [35,75]. L-cysteine is a common amino acid in organisms and is involved in cellular redox networks. L-cysteine is associated with tumor ferroptosis [76], aging [77], etc., and exists in sweat [78]; it potentially can be used as a biomarker for various diseases.

In addition, sweating is also related to some specific disease states, such as amoeba liver abscess, which easily leads to night sweats [79], and up to 75% of menopausal women are affected by night sweats [17].

Thus, the change in sweat components is closely related to health status and chronic diseases. Elevated sweat Cl^–^ concentration is used as the gold standard biomarker for cystic fibrosis; sweat glucose has also attracted numerous studies, and more biomarkers need to be verified through clinical trials. The development of epidermal wearable biosensors to detect biomarkers in sweat will avoid the inconvenience of invasive blood collection and allow continuous, non-invasive, and convenient health monitoring, as well as chronic disease risk warning.

**Table 1:** Clinical uses and potential sweat biomarkers of chronic disease.

Chronic Disease	Sweat Biomarkers	Characteristics	Applications	References
Cystic fibrosis	Cl^−^	≥60 mM	Gold standard in clinical	[30]
Behcet’s disease	l-citrulline l-pyroglutamic acid urocanic acid 2-oxoadipic acid cholesterol 3-sulfate pentadecanoic acid	Needs further exploration	Pre-clinical	[70]
Schizophrenia	Annexin-5 Bleomycin hydrolase Thioredoxin Caspase-14 Synaptophysin	Needs further exploration	Pre-clinical	[72]
Epilepsy	Menthone	A newly described human alarm pheromone	Pre-clinical	[73]
Diabetes	Glucose	[G]_s_/[G]_b_^1^ ≈ 0.01	Pre-clinical	[51,61]
Diabetic ketoacidosis	β-hydroxybutyrate	Needs further exploration	Pre-clinical	[68]

^1^ [Glucose]s_sweat_/[Glucose]_blood_.

## 3. Sweat Sensing Platform

Compared with blood and urine analysis, sweat analysis has many advantages. However, sweat analysis has some challenges too. Health monitoring and clinical diagnosis via sweat can be difficult due to pollution, evaporation, and a lack of real-time sweat sampling and sensing equipment [60]. With the development of sensors for continuous monitoring of sweat [80,81,82] and the development of multiple sensor arrays for real-time analyte detection [22,83], sweat sensing is becoming a technology that can use a non-invasive platform to provide access and monitoring for continuous analyte, attracting more researchers to conduct studies in this field.

### 3.1. Extraction and Collection of Sweat Samples

#### 3.1.1. Sweat Extraction

- Passive sweat extraction

**Figure 2:**
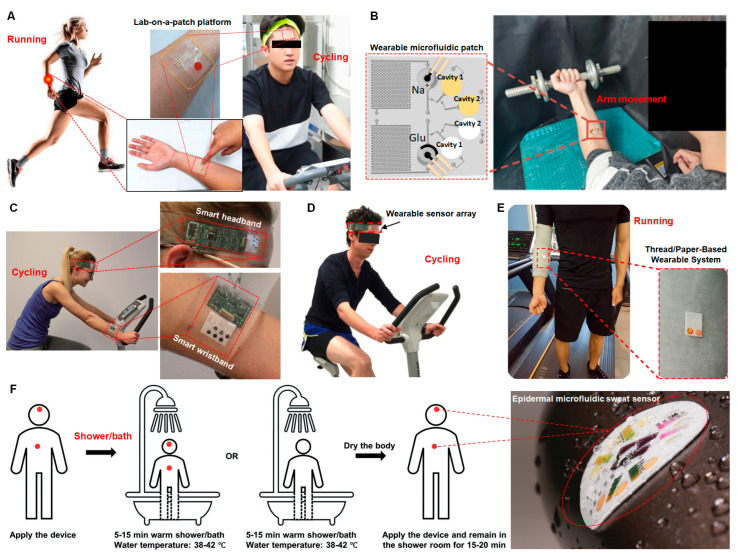
Epidermal wearable biosensors for passive sweat extraction through strenuous exercise (**A**–**E**) and increasing ambient temperature (**F**). (**A**) Subjects exercised by running or cycling while the Lab-on-a-patch platform was attached to their arm or forehead (Adapted with permission from Ref. [87]. Copyright 2020, Elsevier). (**B**) A subject exercised by moving his arm while the wearable microfluidic patch was attached to his arm (Reprinted with permission from Ref. [20]. Copyright 2022, AIP Publishing). (**C**) A subject wearing a ‘smart headband’ and a ‘smart wristband’ during cycling (Reprinted with permission from Ref. [22]. Copyright 2016, Springer). (**D**) A subject exercised by cycling while the wearable sensor array was attached to his forehead (Reprinted with permission from Ref. [89]. Copyright 2016, American Chemical Society). (**E**) A subject wearing an arm guard-integrated device during running (Reprinted with permission from Ref. [84]. Copyright 2020, Springer). (**F**) Schematic illustration of procedures to extract sweat using an epidermal microfluidic sweat sensor during and, or after warm water showering or bathing (Reprinted with permission from Ref. [91]. Copyright 2019, Royal Society of Chemistry).

Passive sweat extraction is a routinely used non-pharmacological strategy for sweat capture. At present, most wearable sweat sensors are based on passive sweat extraction. They are limited to certain conditions, such as strenuous exercise, as shown in Table 2. Usually, these biosensors are made of paper or elastic silicone and put on the skin of the arm or forehead through transparent medical tape or double-sided tape (Figure 2) [20,84,85,86,87,88], since the arm and forehead normally perspire faster than the rest of the body surface. Also, they can be integrated into an arm guard, wristband, or headband (Figure 2) [22,84,89]. Generally, subjects need to do vigorous exercise (running, cycling, or arm movement) for minutes to more than an hour to produce enough sweat, because the sweat rate differs from person to person [87,88]. The detected sweat biomarkers are usually closely related to exercise functions, including glucose, lactate, cortisol, Na^+^, Cl^−1^, K^+^, etc. [20,22,84,85,86,87,88,89,90]. Although studies have proven the feasibility of detecting biomarkers in human sweat under special conditions (strenuous exercise), the sweat secretion rate fluctuates greatly, which affects the accuracy and reliability of the results. It is difficult to detect when the sweat secretion rate is relatively low, such as during long-term sitting and at low temperatures. Also, the method of sweating through exercise is not suitable for patient populations, such as infants and the elderly, nor for the detection of chronic disease markers.

Increasing the ambient temperature to promote sweating is another common way to extract sweat passively; for example, showering and bathing are simple broadly applicable means for sweat extraction (as shown in Figure 2 and Table 2) that can bypass physical stimulation requirements and have been used for chronic kidney screening [91]. However, this passive sweat extraction method limits the ability of the sensor to achieve continuous and long-term real-time monitoring of sweat.

- Active sweat extraction;

Active extraction is needed to continuously collect sweat, such as through iontophoresis and local thermal stimulation that can cause the skin to secrete a lot of sweat [92,93].

**Figure 3:**
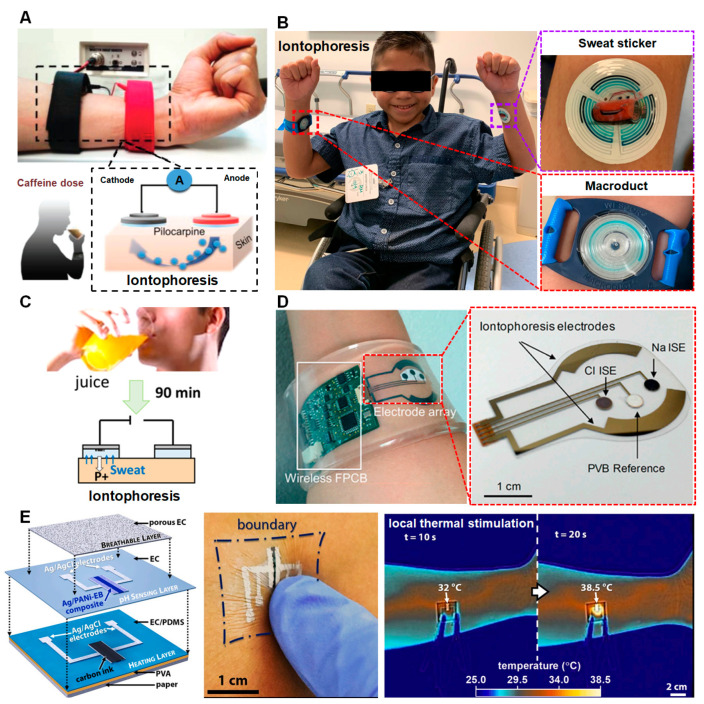
Epidermal wearable biosensors for active sweat extraction through iontophoresis stimulation (**A**–**D**) and local thermal stimulation (**E**). (**A**) Schematic of sweat stimulation by iontophoresis delivery of pilocarpine and caffeine monitoring (Reprinted with permission from Ref. [58]. Copyright 2018, John Wiley and Sons). (B) Based on iontophoresis sweat extraction, a pediatric subject’s sweat Cl^−^ was monitored for a cystic fibrosis diagnosis (Reprinted with permission from Ref. [98]. Copyright 2021, The American Association for the Advancement of Science). (**C**) Schematic of the epidermal biosensor for sweat vitamin C monitoring based on iontophoresis sweat extraction (Reprinted with permission from Ref. [40]. Copyright 2020, American Chemical Society). (**D**) Iontophoresis electrode and sweat sensor electrode [97]. (**E**) Electronic decal containing a heating layer to apply localized heat stress on the skin for rapid sweat production (Reprinted with permission from Ref. [93]. Copyright 2020, Elsevier).

Iontophoresis, also known as ion electrophoresis, is a method that uses continuous direct current to drive ions or charged chemical drugs into the body based on the principle of homoelectric repulsion. In 1959, Gibson and Cooke [94] developed the iontophoresis method; they used the cationic drug pilocarpine to produce sweat. A short-term electrical stimulation can deliver secretory agonist molecules to stimulate sweat glands to produce continuous sweat for several hours [16,95]. The use of iontophoresis to induce sweat production can realize the real-time active extraction of sweat, which solves the difficult problem of real-time monitoring of sweat under natural conditions [81]. The sweat obtained by this method is also widely used in clinical research [96]. Current research mostly uses iontophoresis to extract sweat and monitor sweat through laboratory equipment [58]. Emaminejad et al. combined this idea with a wearable sensing unit and designed an electrochemically enhanced iontophoresis interface to realize a sweat stimulation-sampling-sensing integrated sensing system, which can extract enough sweat volume for stable analysis without causing patient discomfort (as shown in Figure 3) [97]. The system can be programmed to periodically induce sweat secretion and have different secretion characteristics (as shown in Table 2). Therefore, it can be used to obtain a previously unachievable observation of the secretion process to advance our understanding of sweat gland physiology. By integrating this capability, a fully integrated and autonomous platform is formed and demonstrated that can stimulate sweat secretion and can monitor the collected sweat analytes (such as glucose, Na^+^, and Cl^−^). This research expands the targets of sweat health monitoring and further plays a role in disease diagnosis. In addition, the research group also studied the effects of different drugs (acetylcholine or methacholine or pilocarpine, etc.) and different doses on the time and rate of sweat production to further control the rate and total amount of sweat extraction.

In addition, local thermal stimulation can also produce sweat in an efficient and controllable way. Pal et al. [93] fabricated an electronic decal containing a resistive heating element that can apply localized heat stress (38.5 °C for 3 min) on the skin to stimulate rapid production of sweat (180 μL/cm^2^/hour) (as shown in Figure 3E and Table 2).

Compared to the passive sweat extraction method, the active sweat extraction method using iontophoresis and local thermal stimulation has various advantages: First, the active method can realize the control of sweat secretion, which improves the ability of in situ and real-time detection of sweat and the reliability of detection results [40,93,95,98]. Second, sweat stimulation can be utilized without strenuous exercise, which is very important for monitoring the health or disease status of patients, the elderly, and infants [98]. Third, these methods can greatly enrich the types of sweat biomarkers detected and expand the application of wearable sweat biosensors, such as the detection of sweat Cl^–^ for cystic fibrosis diagnosis and management [98], the dynamic monitoring of vitamin C for non-invasive nutrition status assessments toward detecting and correcting nutritional deficiencies [40], and the non-invasive and continuous point-of-care drug monitoring and management [58]. It is the development direction of sweat extraction research. However, the research of active sweat extraction methods is still in the preliminary exploration stage, and its core mechanisms (such as the relationship between electric current, agonist agents, and sweat secretion rate when using iontophoresis) and the stability of long-term work, etc., needs to be further studied.

**Table 2:** Passive and active sweat extraction methods.

Sweat Extraction Methods	Parameters	Lasted Time	Detected Biomarkers	Reference
**Passive sweat extraction**				
Running	Not provided	10, 25, 40 min	lactate, pH	[84]
Running	① 4 km/h for 2 min ② 8 km/h for 5~20 min ③ 3 min cooling down	10, 15, 20, 25 min	glucose, lactate, pH, Cl^−1^	[85]
Running	① 10 km/h for 10 min ② 15 km/h for 10 min ③ 5 km/h for 10 min	30 min	glucose, lactate	[86]
Arm movement	Not provided	60 min	Na^+^, Cl^−1^, pH, glucose,	[20]
Cycling	Not provided	15–40 min	cortisol	[87]
Cycling	① Ramp up for 10 min ② medium-high activity for 30–70 min ③ cooling down for 10 min	50–90 min	pH, glucose	[88]
Cycling	① Ramp up for 10 min ② cycling for 20 min ③ cooling down for 3 min	33 min	Glucose, lactate, Na^+^, K^+^	[22]
Cycling	① Ramp for 5 min ② cycling for 20 min ③ cooling down for 5 min	30 min	Ca^2+^, pH	[89]
Shower/Bath	Water temperature: 38–42 °C	5–15 min	Creatinine, urea, pH	[91]
**Active sweat extraction**				
Iontophoresis	Pilocarpine ^1^	5 min	Cl^–^	[98]
Iontophoresis	Pilocarpine ^1^	10 min	Vitamin C	[40]
Iontophoresis	Pilocarpine ^1^	5 min	Glucose	[95]
Iontophoresis	Pilocarpine ^1^	5 min	Caffeine	[58]
Iontophoresis	Pilocarpine, acetylcholine, methacholine ^1^	5 min	Na^+^, Cl^−^, glucose	[97]
Local heating	38.5 °C ^2^	3 min	pH	[93]

^1^ Agonist agent for iontophoresis. ^2^ Local heating temperature.

#### 3.1.2. Sweat Collection

Microfluidic technology has been rapidly developed in recent years [99]; due to its excellent stretch ability, biocompatibility, and high accuracy, it has become a model of new analysis tools for wearable biosensors [100]. The introduction of microfluidic technologies to the sweat sensor has provided a good solution for the in situ collection of sweat and makes the sweat collection process easier [7,101,102,103]. The microfluidic system can indeed be integrated into body fluid sampling for real-time and continuous monitoring methods [5], and several passive and active sweat collection methods have been developed.

- Passive sweat collection

Passive sweat collection refers to the driving and transportation of sweat from the skin to the sweat collection zone or sensors based on the pressure from the eccrine gland or the capillary action generated by the microfluidic channels or cotton thread or cotton fabric.

**Figure 4:**
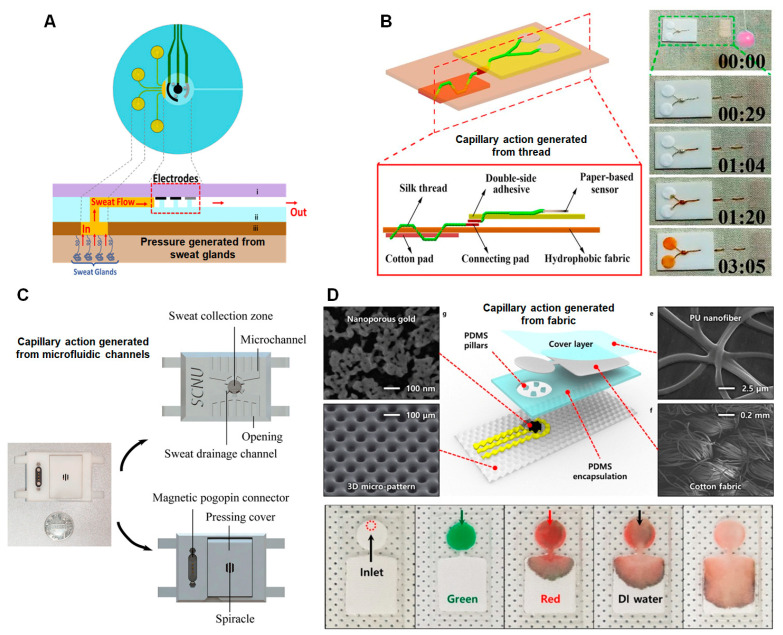
Epidermal wearable biosensors for passive sweat collection. Sweat collection through (**A**) pressure generated from sweat glands (Adapted with permission from Ref. [105]. Copyright 2017, American Chemical Society) and capillary action generated by (**B**,**D**) cotton thread or fabric (B is adapted with permission from Ref. [84]. Copyright 2020, Springer; D is reprinted with permission from Ref. [101]. Copyright 2019, American Chemical Society) or (**C**) microfluidic channels (Reprinted with permission from Ref. [106]. Copyright 2021, Elsevier).

Many research groups use the natural pressure generated by sweat glands (~3 kPa) to drive sweat sampling and combine it with a microfluidic system to collect sweat in situ, as shown in Figure 4A [7,88,95,98,104,105]. Since the internal volume of a microfluidic channel is approximately several microliters, adequate pressure from the eccrine glands could provide continued and passive sweat flow during perspiration [88], taking several minutes to fill the collection area, independent of any need for external force.

Capillary action is also the main internal driving force for sweat collection. For commonly used microfluidic channels with a small size (e.g., diameter < 0.1 mm), the sweat could overcome the positive capillary pressure generated by the hydrophobic channel wall, and enter the microchannel for capture and collection, as shown in Figure 4C [93,106,107]. Besides, the cotton thread or fabric is very hydrophilic after its natural wax is removed and can also effectively produce capillary action to wick sweat from the skin surface, delivering the sweat to the sensors in several minutes, as shown in Figure 4B,D [84,85,101,102].

In short, with the optimization of materials and structures, microfluidic technology can become an excellent, rapid, and low-cost sweat collection platform for wearable biosensor applications, but there are challenges in the accuracy and effectiveness of next-generation wearable microfluidic biosensors. First, the collected sweat volume fluctuates greatly, which affects the accuracy and reliability of the sensing results. Second, sweat evaporation is unavoidable, which results in the enrichment of analytes, thus leading to concentration miscalculation. Third, contamination in sweat collection cannot be ignored, resulting in systematic and random errors.

- Active sweat collection;

The current research trend focuses on the miniaturization of sweat sensors in flexible microfluidic devices to allow efficient sweat collection and minimize external contamination for sweat biomarker detection. The structures of the microfluidic channels have been greatly improved, and the innovation and optimization focus on the integration of passive or active pumps or valves for efficient and controllable sweat collection, such as hydrogel pumps [108,109], super hydrophilic microchannel pumps [20,86,107], hydrophobic valves [107], capillary bursting valves [20,100,110], flap valve [87], microheater-controlled thermo-responsive hydrogel valves [111], etc.

**Figure 5:**
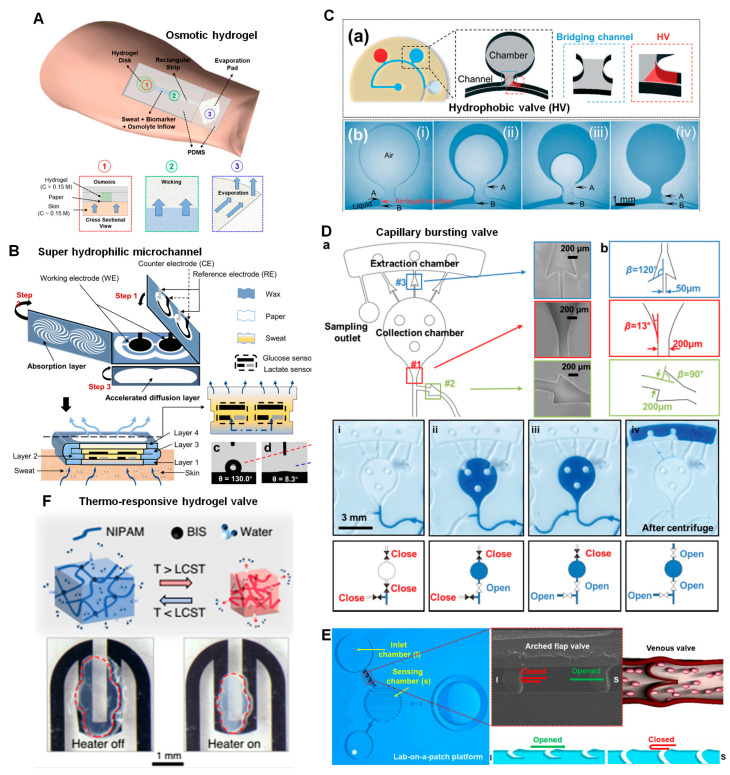
Epidermal wearable biosensors for active sweat collection. The structures of the microfluidic channels have been greatly improved for efficient and controllable sweat collection, such as (**A**) hydrogel pumps [108], (**B**) super hydrophilic microchannel pumps (Reprinted with permission from Ref. [86]. Copyright 2021, Elsevier), (**C**) hydrophobic valves (Reprinted with permission from Ref. [107]. Copyright 2020, Royal Society of Chemistry), (**a**) Schematic of the one-opening chamber with the hydrophobic valve and a bridging channel in a microfluidic device, (**b**) Optical images (i–iv) of the hydrodynamic flow process into the one-opening chamber with a hydrophobic valve, (**D**) capillary bursting valves (Reprinted with permission from Ref. [110]. Copyright 2017, Royal Society of Chemistry), (**a**) detailed schematic illustration of a unit cell in a device, including a collection chamber, extraction chamber, sampling outlet, and three capillary bursting valves and SEM images of the capillary bursting valves, (**b**) sketch of capillary bursting valves with indicated channel width and diverging angle, and optical images and schematic illustrations of the working principle of the capillary bursting valves for chrono-sampling (i–iv). (**E**) flap valve (Adapted with permission from Ref. [87]. Copyright 2020, Elsevier), and (**F**) micro-heater-controlled thermo-responsive hydrogel valves [111].

A hydrogel pump that combines osmosis (hydrogel) and capillary action (microfluidic channels) could realize long-term sweat-wicking (Figure 5A) [108,111]. The hydrogel, infused with a highly concentrated solute (osmolyte) generates the osmotic driving force with the skin, sucking the sweat into the detection zone until the chemical potential difference between the skin and the hydrogel is maintained [108]. The significant advantage of a hydrogel pump is that it can provide long-term operation (from tens of minutes to several hours) without any external power source.

Hydrophilic microchannel pumps are also commonly used for efficient sweat collection. The hydrophilic microchannels that are associated with the positive driving effect of capillary force could spontaneously wick sweat into the microchannels rapidly [107]. Constructing a more hydrophilic channel surface by arranging the microcolumn arrays in the channel in this way could provide a larger contact area for the capillary force and achieve a longer driving effect [20]. Also, printed hydrophilic paper folded into a multi-layer structure could form a reasonably designed three-dimensional (3D) sweat diffusion path, providing an efficient pathway for long-term sweat collection (Figure 5B) [86].

Microvalves, such as hydrophobic valves, capillary bursting valves, arched flap valves, and thermo-responsive hydrogel valves, are designed for effectively guiding sweat flow direction. The hydrophobic valve is a specific zone in the hydrophilic microfluidic channel for sweat flow modulation. After exposing the surface to air plasma treatment, the PDMS microfluidic channel becomes hydrophilic, while the hydrophobic valve can be simply created using a mask to avoid exposure of the PDMS to the air plasma. The pressure induced by the sweat glands drives flow through the microchannel, and the hydrophobic valve helps route sweat into the detection chamber and then allows sweat flow after the chamber is completely filled (Figure 5C) [107]. The significant advantage of the hydrophobic valve is that it reduces sweat sample evaporation and contamination because evaporation and contamination mainly occur through the outlet of microchannels. Additionally, if the hydrophobic valve is combined with multiple chambers, it allows for the sequential generation of sweat collection at different times for long-term analysis [107]. The capillary bursting valve is a specific microstructure in a microfluidic channel for passively guiding sweat through the channel in a sequential fashion (Figure 5D) [20]. Bursting valves designed with different parameters can be opened at different pressures induced by the sweat glands, thereby providing a precise sampling capability [100]. The arched flap valve, inspired by the structure and function of venous valves in human veins, is another specific microstructure to guide sweat flow in a one-way manner (Figure 5E) [87]. The thermo-responsive hydrogel valve incorporated into the microfluidic system is controlled by a microheater and can render active sweat management (Figure 5F) [111]. When the hydrogel’s temperature is lower than its lower critical solution temperature (LCST), its structure expansion and sweat flow are completely blocked with no leakage (off-state). Conversely, when the hydrogel’s temperature is higher than its LCST, its structure shrinks and sweat flow is permitted (on-state). Thus, the thermo-responsive hydrogel valve is an ideal binary off or on valve, by which the sweat collection can be controlled actively.

In short, a hydrogel pump and a super hydrophilic microchannel pump can efficiently promote sweat collection, while the hydrophobic valve, capillary bursting valve, flap valve, and microheater-controlled thermo-responsive hydrogel valve can realize sweat collection in a controllable manner. The significant advantages of various valves for sweat collection are as follows: (1) Valves incorporated at the junction of the chamber and microfluidic channel can reduce sweat evaporation through the outlet of the microfluidic channel. (2) Reduce the unnecessary contamination of sweat, which is vital for accurate sweat analysis. (3) Passive valves (e.g., hydrophobic valves, capillary bursting valves, or flap valves) without active components or power sources present an attractive option for fluid control. (4) Active valves (e.g., thermo-responsive hydrogel valves) can realize fluid control in a flexible and programmable manner.

### 3.2. Fundamentals of Biosensors

#### 3.2.1. Electrochemical Biosensors

Electrochemical biosensors are the most commonly used methods for sweat biomarker detection (glucose, lactate, cortisol, cytokines, nutrients, etc.) where analytes are converted into electrical signals by functionalized electrodes and then transmitted to the processing components [38,40,80,104,112,113]. Based on the different mechanisms of detecting electrodes and identifying the analytes, the electrochemical biosensors can be categorized into three main types, including enzyme-based [80,114,115,116], immune-based [92,101,117], and aptamer-based biosensors [37,118,119].

- Enzyme-based biosensors

The enzyme-based electrochemical biosensors detected by amperometry or voltammetry mainly include biological recognition elements and enzymes, which can selectively catalyze the analyte in sweat and generate electrical signals at enzyme-electrode interfaces; the electrical signals are related to the analytes’ concentration [40,96,120]. Therefore, electron transfer at the enzyme-electrode interface is the key step for such wearable electrochemical biosensors [121]. At present, three generations of enzyme-based electrochemical biosensors based on different electron transfer strategies have been developed [114,116,117,121,122,123,124].

**Figure 6:**
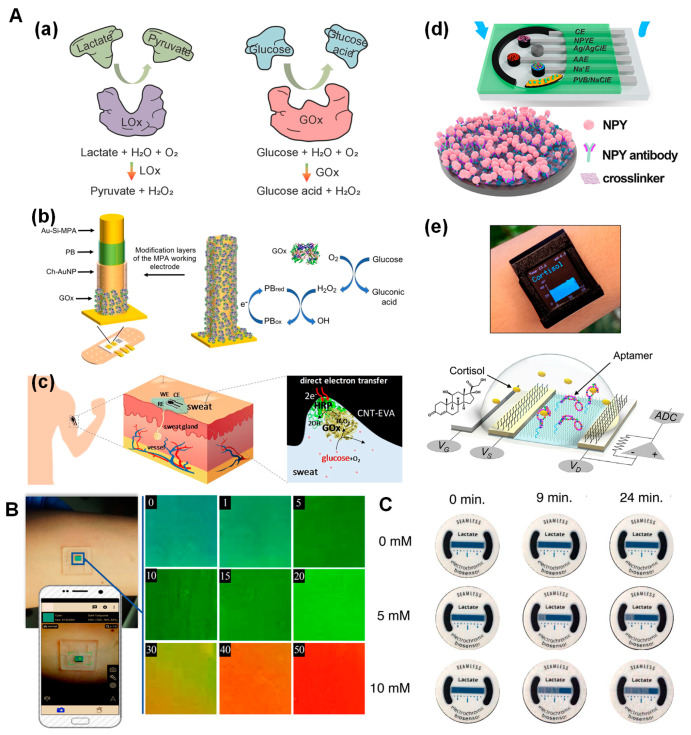
Fundamentals of epidermal wearable biosensors. (**A**) Electrochemical biosensors, (**a**) first− (Reprinted with permission from Ref. [114]. Copyright 2022, Elsevier), (**b**) second− (Reprinted with permission from Ref. [129]. Copyright 2022, American Chemical Society), and (**c**) third−generation (Reprinted with permission from Ref. [121]. Copyright 2020, Elsevier) enzyme-based biosensors, (**d**) immune-based biosensors (Reprinted with permission from Ref. [117]. Copyright 2022, American Chemical Society) and (**e**) aptamer-based biosensors [119]. Colorimetric biosensors use (**B**) a cell phone (Reprinted with permission from Ref. [151]. Copyright 2020, American Chemical Society) or (**C**) the naked eye (Adapted with permission from Ref. [150]. Copyright 2021, Elsevier) to capture the color changes.

In the first-generation biosensor, the enzyme (e.g., lactate oxide, and glucose oxidase) is immobilized on the surface of the working electrode through a crosslinker (e.g., dithiobis succinimidyl propionate). Lactate detection in eccrine sweat is achieved by the catalytic oxidation of L-lactate into pyruvic acid and H_2_O_2_ through lactate oxide (LOx) [116], whereas glucose detection is achieved by the catalytic oxidation of glucose into gluconic acid and H_2_O_2_ through glucose oxidase [114], as shown in Figure 6A. Because the redox-active centers of enzymes are often deeply embedded by polypeptides, electron transfer between enzymes and electrodes at enzyme-electrode interfaces is usually not easy to achieve, leading to low sensitivity [125,126].

In the second-generation biosensor, electron mediators are used to enhance the enzyme electrochemical reactions and to achieve efficient electron transfer from the enzyme’s activity center to the electrodes during sensing applications [127,128], resulting in better electrochemical performance. Carbon quantum dots (CQDs) and Prussian blue (PB) with high electron transfer rates are most commonly used to promote electron transfer and reduce interfacial impedance on the electrode surface (Figure 6A) [106,117,129,130,131,132,133,134]. However, the use of electron mediators not only increases the complexity of biosensors but also leads to low selectivity and instability due to cross-reactions and leakages of the electron mediators, respectively [121,135].

In the third-generation biosensor, direct electron transfer at the enzyme-electrode interfaces without using electron mediators with high selectivity and sensitivity is developed. For example, Xia et al. constructed a flexible and hierarchical meso- or macroporous carbon nanotubes (CNT)—ethylene-vinyl acetate copolymer (EVA) film as the sensing substrate [121]. Due to its well-formed three-dimensional conductive nanoporous structure, direct electron transfer-type bioelectrocatalysis is successfully realized on the glucose oxidase-horseradish peroxidase bienzyme functionalized CNT-EVA film for glucose monitoring without any electron transfer mediator (Figure 6A) [121].

- Immune-based biosensors

The immune-based electrochemical biosensors can detect proteins or small molecules using an affinity-based biosensing scheme with an antibody as a recognition element (Figure 6A) [38,87,104]. Due to the highly specific recognition properties of antibodies to antigens, immune-based electrochemical biosensors usually have excellent selectivity and specificity. The electrochemical impedimetric spectroscopy (EIS) and differential pulse voltammetry (DPV) techniques are commonly used for electrical signal detection [38,87,100,104], and the electrical signals (e.g., impedance, and capacitance) are dependent on the antibody-antigen binding activity [117].

- Aptamer-based biosensors

Aptamers are short single-stranded oligonucleotides (SSDNA or RNA) that are typically made of 20–80 nucleotides with stable tertiary structures to specifically bind the target [119,136,137]. Aptamer-based electrochemical biosensors have gained considerable attention because of their rapidity and accuracy in terms of quantification of a vast variety of targets, including macromolecules, small molecules, bacteria, and cells, with high affinity and specificity [37,118,137]. The aptamer probes anchored on substrates (e.g., graphene) can recognize and capture the specific biomarker via a chemical bond (Figure 6A); the specific binding between the aptamer and biomarker induces a change in electrical signals, which is detected, determining the biomarker concentration [36,37,138,139,140,141].

The aptamer-based biosensors show significant advantages compared with immune-based biosensors: (1) Aptamers are chemically synthesized biorecognition elements with high selectivity and affinity, making them adaptable to multiple targets at low concentrations [142,143,144,145]. (2) Aptamers are smaller in size than their antibody counterparts, thus allowing for high-density mounting on a given effective surface area. This type of mounting makes them reliably capture analyte molecules for prolonged times without any saturation [146,147,148]. (3) Aptamers exhibit thermal and chemical stability, causing easier conjugation to a substrate and long-term stability [37,139].

#### 3.2.2. Colorimetric Biosensors

Colorimetric biosensors use electronic equipment (e.g., the camera of a cell phone [85,149], or the naked eye [150]) to capture the sensor’s color changes in response to analytes (Figure 6B,C) and then perform an image analysis for the analytes’ quantification [7,80,81,101,151,152,153]. The color changes of the sensing parts are commonly based on various mechanisms, including the presence of specific analytes, red-shift reflected color of photonic interpenetrating polymer network film [151], fluorescence resonance energy transfer [154], peroxidase-mediated oxidation of 3,3′,5,5′-tetramethylbenzidine (TMB) [149], Raman spectroscopy [90], Prussian Blue [150], etc. The signal detection scheme of colorimetric epidermal wearable biosensors is particularly useful for the patient’s daily health self-monitoring.

### 3.3. Substrates of Biosensors

Epidermal wearable biosensors have been manufactured on a variety of substrates, such as textiles [114,123,124,152,155,156], tattoos [40,80,81,93,157], and patches and bandages [149,151,158,159,160,161,162,163,164,165,166,167]. 

Yoon et al. coated the surface of the CFT (carbon fiber thread) electrode with a layer of citric acid supramolecular polymer that can self-heal within 30 s at room temperature, which is highly sensitive and self-healing [155]. The sweat thread is woven into textiles and integrated with a wireless FPCB (flexible printed circuit board) to realize real-time monitoring of sweat, and it can run normally even after being cut for about 20 s.

Due to the unique mechanical physiology of human skin, researchers have focused on developing a tattoo platform that closely contacts the wearer’s skin and mimics the characteristics of the skin. At present, tattoo-based sweat pH sensors, Na^+^ sensors, NH_4_^+^ sensors, and lactic acid sensors that detect analyte activity by voltammetry have been developed [80,81]. Potential sensors generally include indicator electrodes and reference electrodes. For example, Bandodkar et al. made carbon and Ag or AgCl into two “eyes-shaped” indicator electrodes and reference electrodes, made the contact pad into a two “ears” form, and finally made the sweat pH sensor into the “smiley face” style and applied it to real-time monitoring of human sweat pH [157]. The results showed that this type of tattoo sensor can withstand the mechanical deformation of human skin during exercise. Therefore, the concept of the tattoo-ion-potentiometer sensor can be extended to monitoring other clinically relevant sweat markers. This type of sensor is ergonomic and supports direct and continuous contact with the skin surface, making it a unique platform for wearable sensors. However, when applied to continuous monitoring, it is necessary to consider the impact of daily skin cleaning on the performance of the sensor device.

In recent years, sweat sensor patches and bandages similar to smart tattoos have also been used to continuously monitor sweat biomarkers. Bae et al. reported a fully integrated stretchable microfluidic glucose sensor patch that integrates an omnidirectional stretchable NPG (Nanoporous gold) electrode on a 3D micro-patterned PDMS (Polydimethylsiloxane) substrate with stress absorption. The integrated glucose sensor patch shows an excellent ability to continuously and accurately monitor sweat glucose levels [101]. Huang et al. made a high-polymer patch sensor to detect the pH value of sweat and the colorimetric analysis of metal ions [153]. Rose et al. reported an RFID (radio-frequency identification) bandage-based sensor. They used ion selection to detect Na^+^, K^+^, Mg^2+^, and NH_4_^+^ plasma in sweat sequentially [92]. Sweat sensor patches and bandages will play an important role in the medical field due to their convenience, but patch sensors are usually thick and have limited stretch ability, and are not suitable for a wide range of applications on human skin. In contrast, the bandage sensor has better ductility and a better fit with human skin, but its commercial use still needs further research and optimization.

## 4. Clinical and Preclinical Applications

Sweat is widely used in epidermal biosensors to provide useful information for continuous monitoring due to its multiple sampling locations, continuous collection, easy and comfortable placement of the collection device, and rich physiological information [22,67,81,166,168].

In the traditional clinical environment, blood, and urine samples are mainly used for routine analysis. These techniques are costly, time-consuming, and cannot provide continuous measurement of target analyte concentration [60]. Clinical biosensors can directly measure the concentration of biomarkers in body fluids in a real-time and continuous manner without the need for centralized laboratory facilities. This ability will reduce the need for frequent sample collection and may open up new avenues for biomarker-directed therapeutic intervention. There are high requirements for the sustainability and effectiveness of biosensors in clinical applications. Passive sweat collection using multiple capture antibody microarray technology [169] and active sweat induction based on iontophoresis [97] are currently being developed to improve the durability of the sensor. Wearable biosensors dedicated to clinical diagnosis are mainly based on potential, ampere, and voltametric measurements [156].

Biosensing technology has the potential to replace a variety of clinical environments: emergency diagnosis of acute diseases [170], long-term monitoring of relevant physiological indicators of chronic diseases [171], and self-preliminary diagnosis [172] in the case of a lack of medical personnel in the outbreak of disease, namely, physiological information monitoring and clinical disease diagnosis [173].

Physiological information monitoring is of great significance to the diagnosis and treatment of clinical diseases [87,104,164,174]. At present, many wearable sweat sensors for physiological information monitoring have been developed (Table 3). This sensor integrates physiological indicator detection technology with wearables, which can obtain basic physiological indicators at various states [155,175,176,177,178]. With the development of sensor technology and people’s increasing attention to their own health conditions, more and more physiological monitoring equipment is being developed [179,180,181]. Wearable technology can be used to establish a wearable physiological monitoring system to realize the continuous and long-term monitoring of physiological information in patients.

In addition, wearable sweat sensors have also been used in the diagnosis of clinical chronic diseases. Raiszadeh et al. have applied proteomics research to sweat, showing that human skin is the most abundant source of sweat protein and that the human skin has strong proteolytic enzyme activity [72]. Adewole et al. studied the sweat composition of patients with active tuberculosis and detected 26 disease-specific sweat proteins [182]. This study classified high-risk subjects and shortened the time to diagnosis. Katchman et al. detected immunoglobulins in sweat [49] and other researchers demonstrated the correlation between different cytokine concentrations in sweat and serum, indicating that sweat analysis has the potential to be a non-invasive inflammation monitoring tool [53]. This research provides a new method for the clinical detection of related diseases. In addition, wearable biosensors can also diagnose some local skin diseases [183,184] and detect various pharmacological metabolites in sweat [185,186], which can be applied in forensic environments.

It can be seen that researchers can obtain a lot of important body-related data from sweat, which shows the application prospects of wearable sweat sensors in medical and health fields such as disease diagnosis and health management. The establishment of these wearable sweat sensor platforms has brought new development ideas to real-time monitoring—in situ diagnosis—precise treatment personalized medical and health field.

**Table 3:** Epidermal wearable biosensor-based sweat biomarker analysis in clinical and preclinical applications.

Biomarkers	Related Chronic Disease	Substrate of Biosensors	Sweat Extraction Strategies	Sweat Collection Strategies	Sensing Techniques	Detection Range	Detection Limit	Application	Reference
Cl^−^	Cystic fibrosis hyponatremia	Patch	Iontophoresis	Pressures created by sweat glands	Colorimetric	23–100 mM	–	In clinical	[98]
		Textile	Running	Capillary action generated by cotton thread	Colorimetric	10–100 mM	5 mM	Preclinical	[85]
Glucose	Diabetes	Textile	Running	Capillary action generated by cotton thread	Colorimetric	10–250 μM	7 μM	Preclinical	[85]
		Patch	– ^1^	– ^1^	Electrochemical	0.1 nM–1 μM	0.1 nM	Preclinical	[112]
		Patch	Running	Pressures created by sweat glands	Electrochemical	2.38–14.29 mM	3.84 μM	Preclinical	[122]
		Patch	– ^1^	– ^1^	Electrochemical	0.1–0.6 mM	3 μM	Preclinical	[121]
		Patch	–	–	Electrochemical	5 μM–6 mM	1 μM	Preclinical	[134]
		Patch	Cycling	Absorbent patch	Electrochemical	25–150 μM	–	Preclinical	[88]
		Patch	Cycling	Super hydrophilic 3D sweat diffusion path	Electrochemical	0.08–1.25 mM	17.05 μM	Preclinical	[86]
		Patch	Cycling	Capillary action generated by filter paper	Electrochemical	0–1 mM	4.95 μM	Preclinical	[106]
		Textile	– ^1^	– ^1^	Electrochemical	0.01–100 mM	301 nM	Preclinical	[124]
		Patch	–	–	Electrochemical	50 nM–1.07 mM	0.05 μM	Preclinical	[187]
		Patch	– ^1^	– ^1^	Electrochemical	50 μM–1.4 mM	26 μM	Preclinical	[129]
		Patch	Cycling	Pressures created by sweat glands	Enzymatic biofuel cells	0–150 μM	–	Preclinical	[188]
		Patch	Iontophoresis	Pressures created by sweat glands	Electrochemical	1–3243 μM	0.85 μM	Preclinical	[132]
		Patch	Cycling	Pressures created by sweat glands	Electrochemical	0.003–1.5 mM	7 μM	Preclinical	[189]
		Patch	Cycling	Pressures created by sweat glands	Electrochemical	0–1.5 mM	0.025 μM	Preclinical	[190]
Lactate	Fatigue, Pressure ischemia, Insufficient oxidative metabolism	Textile	Running	Capillary action generated by cotton thread	Colorimetric	1.0–12.5 mM	0.4 mM	Preclinical	[85]
		Patch	Running	Pressures created by sweat glands	Electrochemical	2–15 mM	2.61 μM	Preclinical	[122]
		Patch	–	–	Electrochemical	1–600 μM	–	Preclinical	[134]
		Patch	Cycling	Super hydrophilic 3D sweat diffusion path	Electrochemical	0.3–20.3 mM	3.37 μM	Preclinical	[86]
		Patch	Cycling	–	Enzymatic biofuel cells	0–15 mM	–	Preclinical	[188]
		Patch	Cycling	Pressures created by sweat glands	Electrochemical	0–56 mM	4 μM	Preclinical	[190]
		Textile	Running	Capillary action generated by thread	Colorimetric	0–25 mM	0.98 mM	Preclinical	[84]
		Patch	Wear a trimmer belt	Pressures created by sweat glands	Electrochemical	1–100 mM	1 mM	Preclinical	[116]
		Bandage	Cycling	Pressures created by sweat glands	Electrochemical	1–50 mM	32.6 μM	Preclinical	[130]
		Bandage	– ^2^	– ^2^	Electrochemical	0.01–1.35 mM	6.8 μM	Preclinical	[133]
		Textile	Cycling	Capillary action generated by thread	Electrochemical	0–25 mM	3.61 mM	Preclinical	[123]
		Patch	– ^2^	– ^2^	Electrochromic	0–10 mM	–	Preclinical	[150]
		Patch	Exercise	Osmotic hydrogel	Electrochemical	0–15 mM	350 nM	Preclinical	[109]
		Patch	Running	Pressures created by sweat glands	Electrochemical	0.001–25 mM	0.8 μM	Preclinical	[131]
Cortisol	Depression, anxiety	Patch	Cycling	Pressures created by sweat glands	Electrochemical	1 pM–10 μM	10 pM	Preclinical	[118]
		Patch	Arm movement	Pressures created by sweat glands	Electrochemical	1 pM–1 μM	1 pM	Preclinical	[119]
		Patch	– ^3^	– ^3^	Colorimetric	10–1000 ng/mL	6.76 ng/mL	Preclinical	[154]
		Bandage	Normal daily activities	Hydrophilic microporous membrane	Electrochemical	1–256 ng/mL	4 ng/mL	Preclinical	[139]

^1^ A simulated sweat sample containing spiked glucose was used as a test sample. ^2^ A simulated sweat sample containing spiked lactate was used as a test sample. ^3^ A simulated sweat sample containing spiked cortisol was used as a test sample.

## 5. Summary and Prospect

Nowadays, millions of people use precise and affordable fitness trackers and smart watches to monitor physiological indicators such as heart rate and sleep state, which provide certain health assistance and preliminary suggestions. The epidermal wearable biosensor will provide real-time information about the individual’s physiological state at the molecular level, thus transmitting relevant health care or health management information. This type of sensor will lead to technological innovation in the field of wearable sensors. According to research, the wearable sensor market will reach $2.86 billion by 2025. However, from a commercial point of view, the price of wearable epidermal biosensors needs to be lower in order to reach a wider audience. In order to be further marketed, the epidermal wearable biosensor must ensure the stability of the biological receiver and negligible sample contamination.

At the same time, continuous monitoring must be combined with real-time sampling to meet the ultimate clinical application. The range of human biomarkers that can be accurately and continuously monitored by the existing epidermal wearable biosensors is still very limited, and the stability of the sensors and their evaluation through human experiments need to be optimized. Although perspiration can be obtained using non-invasive technology, discontinuity, and skin contamination are still great challenges. Perfect iontophoresis can also cause skin irritation, which is not user-friendly. Therefore, it is necessary to further develop a sweat collection method combining sweat stimulation and microfluidics to realize continuous monitoring. There are also many challenges for ISF sampling and analysis using commercially available devices, such as the biocompatibility of the sensing components and stability of the sensing layer in vivo. Research that solves these challenges should focus on the preparation of sensors from biocompatible materials or the modification of the surface.

Not only can the epidermal biosensor detect biomarkers in sweat, but it can also analyze the biomarkers on the skin’s surface and in ISF. A recently reported bandage biosensor using skin surface tyrosinase as an analyte is an outstanding example of a wearable device designed to detect enzymes as an analyte [191]. The excellent performance of the tyrosinase bandage biosensor shows the prospect of rapid screening for melanoma. If biosensors want to be accepted by the market, a large amount of verification and testing of clinical accuracy is required. Similarly, Trivedi et al. wiped the skin of patients with Parkinson’s disease and separated four kinds of disease-specific proteins from those wipes [192]. Next, Tsunoda et al. successfully used sweat to monitor the process of levodopa treatment for Parkinson’s disease [55]. Such research still needs further study, which will greatly impact the treatment of clinical diseases. In the future, a systematic and in-depth analysis of the composition of each biological fluid will be essential for the innovative development of biomarkers for wearable biosensors.

The current focus of epidermal wearable biosensors is mainly on clinical and health management. Continuous monitoring of disease-related biomarkers through epidermal wearable biosensors can provide useful information on human health. However, one of the challenges lies in the detection of low-abundance biomarkers. However, when combined with the existing microfluidic technology, preconcentration can be one of the solutions. We believe that with the advancement of nanotechnology, and the continuous development of sensor functions, alongside the cooperation of researchers with different backgrounds, this field will gradually be improved and optimized and has great developmental potential.

## Data Availability

Not applicable.
